# Starch Granule Re-Structuring by Starch Branching Enzyme and Glucan Water Dikinase Modulation Affects Caryopsis Physiology and Metabolism

**DOI:** 10.1371/journal.pone.0149613

**Published:** 2016-02-18

**Authors:** Shahnoor S. Shaik, Toshihiro Obata, Kim H. Hebelstrup, Kevin Schwahn, Alisdair R. Fernie, Ramona V. Mateiu, Andreas Blennow

**Affiliations:** 1 Department of Plant and Environmental Sciences, University of Copenhagen, Thorvaldsensvej 40, 1871 Frederiksberg C, Denmark; 2 Max-Planck-Institut für Molekulare Pflanzenphysiologie, Am Mühlenberg 1, 14476 Potsdam-Golm, Germany; 3 Department of Molecular Biology and Genetics, Aarhus University, Forsøgsvej 1, 4200 Slagelse, Denmark; 4 Department of Micro- and Nanotechnology, Technical University of Denmark, Ørsteds Plads, 2800, Lyngby, Denmark; University of Potsdam, GERMANY

## Abstract

Starch is of fundamental importance for plant development and reproduction and its optimized molecular assembly is potentially necessary for correct starch metabolism. Re-structuring of starch granules *in-planta* can therefore potentially affect plant metabolism. Modulation of granule micro-structure was achieved by decreasing starch branching and increasing starch-bound phosphate content in the barley caryopsis starch by RNAi suppression of all three Starch Branching Enzyme (SBE) isoforms or overexpression of potato Glucan Water Dikinase (GWD). The resulting lines displayed Amylose-Only (AO) and Hyper-Phosphorylated (HP) starch chemotypes, respectively. We studied the influence of these alterations on primary metabolism, grain composition, starch structural features and starch granule morphology over caryopsis development at 10, 20 and 30 days after pollination (DAP) and at grain maturity. While HP showed relatively little effect, AO showed significant reduction in starch accumulation with re-direction to protein and β-glucan (BG) accumulation. Metabolite profiling indicated significantly higher sugar accumulation in AO, with re-partitioning of carbon to accumulate amino acids, and interestingly it also had high levels of some important stress-related metabolites and potentially protective metabolites, possibly to elude deleterious effects. Investigations on starch molecular structure revealed significant increase in starch phosphate and amylose content in HP and AO respectively with obvious differences in starch granule morphology at maturity. The results demonstrate that decreasing the storage starch branching resulted in metabolic adjustments and re-directions, tuning to evade deleterious effects on caryopsis physiology and plant performance while only little effect was evident by increasing starch-bound phosphate as a result of overexpressing GWD.

## Introduction

During cereal caryopsis (grain) development, caryopses act as the main sink tissue while the mature leaves, stems or green parts of the ear are the main sites for photosynthesis. The barley caryopsis consists of filial tissues (endosperm and embryo) surrounded by maternal tissue, palea, lemma and the pericarp, the pericarp containing the vascular tissues is the main site for phloem unloading [1]. Transient starch is deposited in the pericarp for a short period from anthesis to 4 days after fertilization (DAF) [2]. It is subsequently re-mobilized after a few days following the disintegration of pericarp cells [3]. The endosperm in the caryopsis is the main site for storage reserve compounds, and starch is the major storage product. Storage starch accumulation usually begins in the wings of the endosperm starting at around 6 DAF and continuing throughout the main grain filling period [2]. Hordein storage proteins accumulate as protein bodies at the same time as starch accumulation [4]. By contrast, deposition of (1,3;1,4)-β-D—glucans (β-glucan (BG) or mixed linkage glucan) begins slightly earlier at about 5 DAP [5].

In cereal caryopsis, storage starch is the primary and most important reserve deposited. Due to the agronomical importance of grains, cereal grains can be considered as bio-factories to improve starch functionality by means of genetic alterations [6–8]. Starch has multiple diverse applications from industrial to health-related benefits [9]. The structure of starch is important for effective mobilization during the grain germination process [10]. The starch metabolic pathway in photosynthetic and non-photosynthetic tissues and its regulation has been extensively discussed [8,11,12] and transcriptional regulation of starch metabolic genes have been documented to be modulated by metabolic intermediates [13]. Starch metabolism has additionally been comprehensively characterized in barley caryopsis development [2,14,15] where it has been demonstrated to exhibit diurnal regulation in developing caryopses [16]. The starch biosynthetic enzymes ADP-glucose pyrophosphorylase (AGPase), starch synthase (SS), starch branching enzyme (SBE), debranching enzyme (DBE), phosphorylase (Pho), and glucan water dikinase (GWD) function together to synthesize the complex starch granule [17].

Plants with deficient isoforms of SBE, which catalyzes the formation of branch points, accumulate less starch [18], with higher amylose content [19,20]. They, therefore, have high resistant starch (RS) content [21], reduced branch point density in amylopectin [22–24] and a generally reduced growth performance [25]. RS is defined as the portion of starch escaping dietary, hydrolytic degradation in the upper gut and to a large extent being fermented in the colon to generate health-associated short chain fatty acids such as butyrate [26]. GWD catalyzes the phosphorylation of starch at C-6 position [27] and the PWD (phosphoglucan water dikinase)/GWD3 homologue phosphorylates starch at the C-3 position of the glucose residue [28,29]. Starch phosphorylation is integrated with starch biosynthesis, despite the fact that its major role appears to be related with starch degradation [17,30]. Our previous studies have indicated that increased starch phosphorylation has a limited direct effect on the efficiency of storage starch degradation rate during germination, but rather seems to induce complex effects on grain metabolism [10].

Alterations in starch biosynthesis could generate changes in carbon partitioning to protein in the caryopsis and hence also in the biosynthetic precursors of central metabolism [31–32]. We have previously demonstrated that endosperm-specific suppression of all the three isoforms SBE I, IIa and IIb in barley grain produced amylose-only starch (AO) with high resistant starch [21] and that endosperm-specific ectopic overexpression of potato GWD in barley produced hyper-phosphorylated cereal starch (HP) [33]. In the present study, we propose that optimized micro-structure of the starch granule is of major importance for normal starch metabolism over caryopsis development. We demonstrate that decreased starch branching has significant effects on caryopsis metabolism and that flexibility of the caryopsis make metabolic adjustments to cope with such alterations. We also demonstrated that hyper-phosphorylation of the starch granule has limited effect on caryopsis metabolism during development. The data are discussed in terms of the premises for metabolic plasticity allowing for specific modification of starch in the cereal grain.

## Results

The Hyper-Phosphorylated (HP) and hyper Amylose-Only (AO) lines were selected from transgenic alleles showing stable phenotypes over several generations. Investigations were performed to evaluate the development of grain constituents, granule morphology and grain/caryopsis metabolism. Different developmental time points were investigated from 10 days after pollination (DAP) to maturity at four developmental stages: 10, 20, 30 DAP and at the mature dry grain stage (MDG, when the grain water content has decreased beneath 10%), to study the grain development phase and the post-desiccation phase (S1 Fig). The main composition of the mature grains is given in Table 1.

**Table 1:** Mature dry grain (MDG) starch, amylose, starch bound C6 phosphate, BG and protein contents of WT and the transgenic lines. Starch, BG and protein contents were determined on the mature dry grains. Amylose and starch bound C6 phosphate was determined on purified starch. Values are presented as means ± SE of five biological replicates.

Line	Starch	Amylose (%in starch)	Beta glucan (% DW)	Protein (% DW)	Grain DW (mg)	Starch bound C6 phosphate (nmol/mg starch)
**WT**	62 (3)	23.5 (0.7)	5.0 (0.3)	8.9 (0.3)	52.4 (1.0)	0.35 (0.02)
**HP**	55 (2)	24.2 (0.4)	5.2 (0.3)	9.4 (0.4)	52.8 (1.7)	3.4 (0.8)*
**AO**	40 (9)**	95.0 (1)**	4.0 (0.4)	11.8 (0.2)**	34.8 (0.9)**	0.50 (0.05)

** denotes significant difference at P <0.001 and

* denotes P <0.01. Grain dry weight values are the means of three biological replicates with a pool of 10 grains each harvested randomly from the entire plant population. Plant growth conditions are described in Materials and Methods.

### Endosperm specific silencing of SBE resulted in decreased starch accumulation and grain dry weight, while the GWD over-expressor remained unaltered

**Fig 1:**
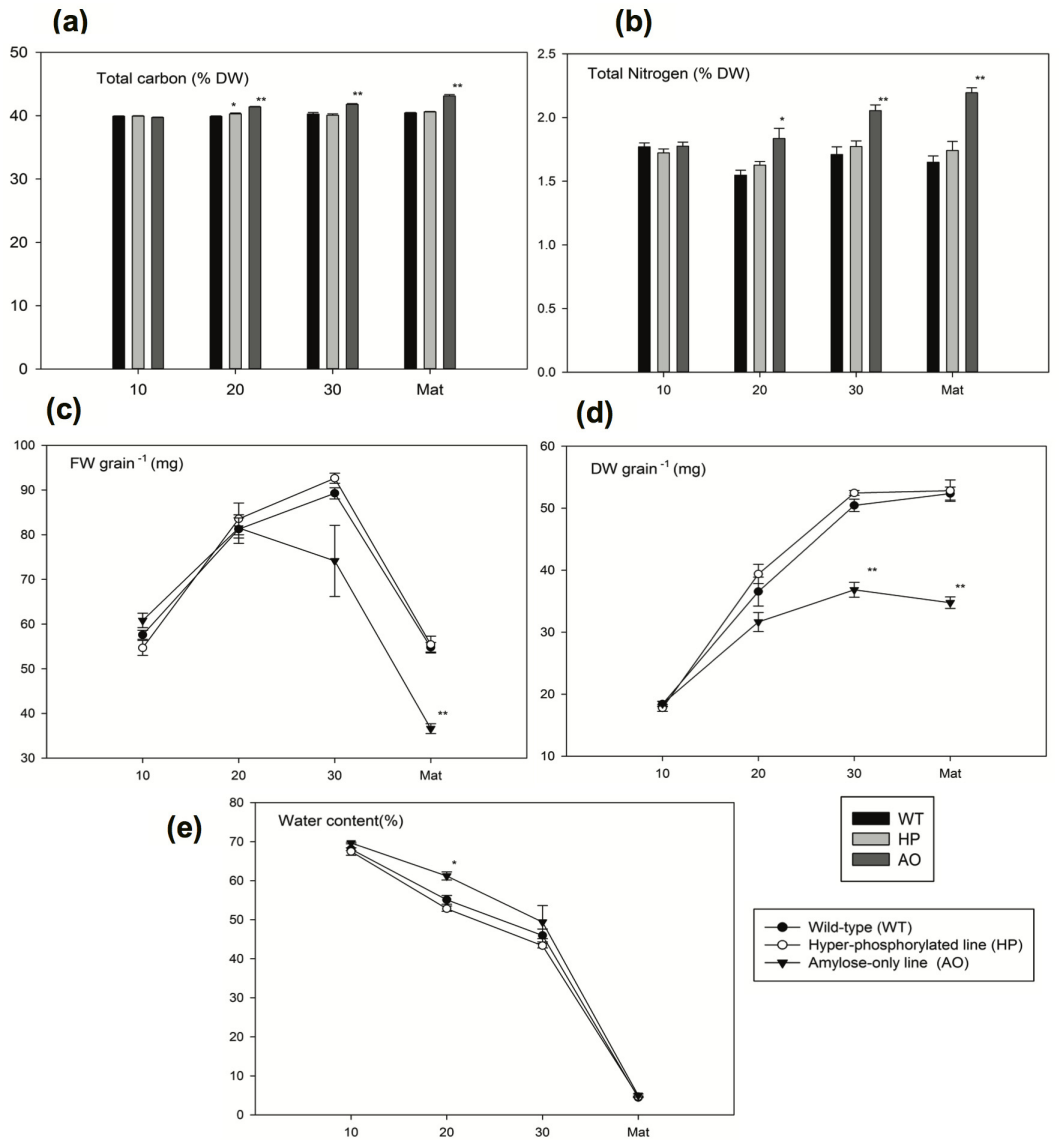
Dry matter accumulation in the developing barley caryopsis. (a) Total carbon and (b) total nitrogen. Values are means of five biological replicates ± SE. (c) Fresh weight (FW), (d) dry weight (DW), and (e) water content in WT, HP and AO at 10, 20, 30 DAP and Mature dry grain. Values are means of three biological replicates ± SE. *Asterisk*s indicate significant differences by one-way anova of transgenic lines compared with the WT at the same developmental stage (* P<0.01, ** P<0.001).

**Fig 2:**
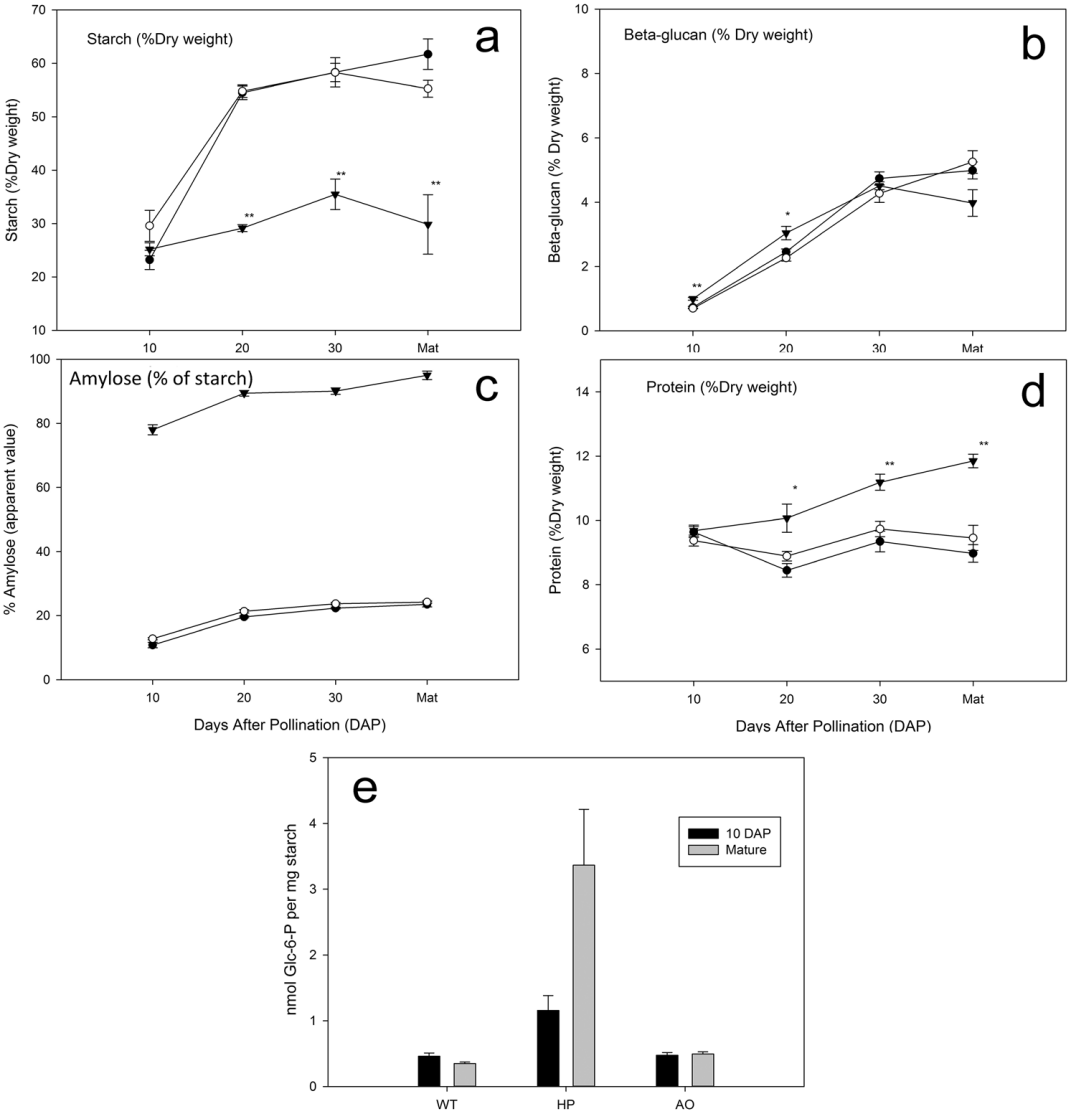
Accumulation of storage products in the developing barley caryopsis. (a) Starch, (b) protein and (c) BG, deposition of starch (d) amylose and (e) phosphate in WT, HP and AO at 10, 20, 30 DAP and Mature dry grain developmental stages. Values are means of five biological replicates ± SE. Asterisks indicate significant differences by one-way anova of transgenic lines compared with the WT at the same developmental stage (* P<0.01, ** P<0.001). For clarity, difference in P value is not indicated where obvious difference is observed. HP: ◯ WT: ● AO:▼.

General grain parameters such as total carbon and nitrogen, fresh weight, dry weight and water content were investigated in wild type (WT), HP (GWD over-expressor grains) and AO (SBE silencing grains). The percentage of total carbon and nitrogen were nearly identical in HP compared to WT except a slightly higher percentage of carbon was observed at 20 DAP, whereas in AO both carbon and nitrogen percentages were significantly higher at 20, 30 DAP and in MDG (Fig 1a and 1b). Grain fresh weight was unaltered in HP and AO as compared to WT at 10, 20, and 30 DAP but AO showed significant difference in the fresh weight in MDG (Fig 1c). The same trend was seen for the dry weight, however, this was significantly lower in AO already at 30 DAP and in MDG but unchanged in HP compared to WT (Fig 1d). The water content in AO was significantly higher at 20 DAP (Fig 1e). Starch content (Fig 2) was also analysed during the same developmental time points. Starch accumulation remained unaltered in HP compared to WT (Fig 2a). This indicates that alterations in the hyper-phosphorylated starch line neither affected starch biosynthesis nor stimulated starch degradation. However, counter-acting carbon flow in starch metabolism cannot be ruled out. AO showed significantly reduced starch accumulation at 20 and 30. AO showed altered storage protein accumulation, which remained unaltered in HP. GWD expression in the HP line is driven by the D-hordein promoter, which is very active already at 12 DAP and continues its activity throughout the time when the vast majority of the starch is synthesized [34].

To investigate whether reduced starch storage in the AO grains affects carbon partitioning to alternative storage compounds, we measured the levels of BG and total protein contents. The deposition of BG was unaffected in HP grains, however, AO showed slightly higher levels at 10 and 20 DAP but ultimately similar levels of BG were deposited at 30 DAP and MDG (Fig 2b). Storage protein accumulation begins at around same time as starch accumulation [14]. HP showed normal rate of storage protein accumulation as WT whilst AO showed significant increase in protein accumulation at 20, 30 DAP and MDG although it showed the same protein level as WT at 10 DAP (Fig 2c). This indicates that the SBE gene suppression in AO affects the storage compound composition towards higher protein content.

### Starch granule amylose and starch phosphate content

We have earlier shown that by separation of amylose and amylopectin using size-exclusion chromatography, the starch in mature dry grains of AO consisted virtually only of amylose (> 99%, [21]. By measuring apparent amylose using iodine staining, we observed that the high amylose content in AO grains increased over the development of the grain (Fig 2c). On the contrary, HP showed similar levels to WT (Fig 2c). The starch bound phosphate levels (C6 phosphate) were measured at 10 DAP and in MDG. HP showed approximately two-fold higher starch bound C6 phosphate at 10 DAP and an approximately ten-fold increase in MDG compared to WT (Fig 2e). No difference in starch bound C6 phosphate content was observed in the AO line compared to WT. Current knowledge, as reviewed in [35] suggests that amylose has very little phosphomonoesters as compared to amylopectin. However, amylose-only plants were not studied. Our study now demonstrates that mainly linear starch can be phosphorylated. This effect can be a result of the low amount of short and clustered chains present in the AO starch (described below) having amylopectin-like segments.

The effects on the branching pattern of the starch in these lines was investigated by chain length distribution analysis at 10 DAP and in MDG. The average degree of polymerization (DP) of the linear chains generated after enzyme-catalysed debranching of the starch in MDG samples of AO was 28.1 compared to 21.7 and 22.3 in WT and HP, respectively (S2 Fig). It should be noted that the chain-length data are normalised to equal area for the three lines and the average numbers take into account only the branched part of the starch being only 8.6% of the WT and HP total areas. Hence, the AO starch is a lightly branched starch just as normal amylose [36]. In conclusion, the two main traits conferred in the different transgenic lines, i.e. amylose content in the AO line and the phosphate content in the HP line, gradually developed and increased over caryopsis development.

### Starch granule size distribution

To investigate any effects of the alterations of SBE and GWD on starch granule size distribution, we measured the granule size distribution in the MDG samples (S3 Fig). The starch granule distributions for the three lines were very similar and the only tendency is a somewhat wider distribution for the AO starch granule population. Bimodal granule size distribution was found as for WT and HP showing two populations i.e., A-type starch granule (> 10 μm) and B-type starch granules (< 10 μm). Since the granules from the AO line are compound, as judged from microscopy, the distribution of these granules cannot be interpreted as distribution of single granules or A-, and B-type granule populations.

### Microscopic structures of starch granules

The purified starch granules at different developmental stages were used for microscopic investigations in order to study the effects of AO and HP starch structures on granule formation.

#### Starch granule micro and nano topography

**Fig 3:**
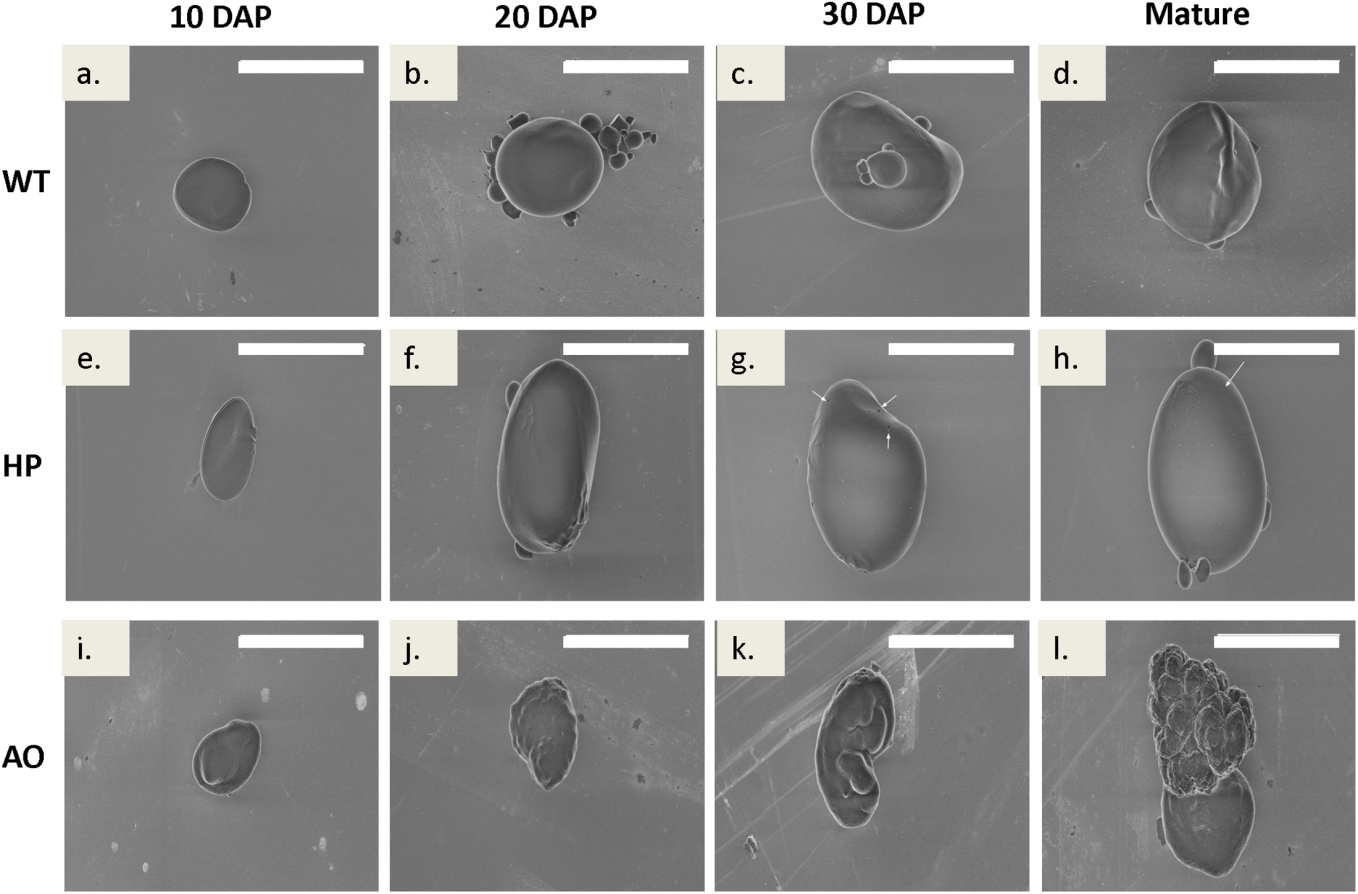
Single starch granule topography and morphology of selected typical specimens analysed by non-coated scanning electron microscopy. Starch purified from developing caryopsis of WT (a-d), HP (e-h) and AO (i-l) at 10, 20, 30 DAP and mature dry grain developmental stages. Red arrows indicate rare surface pores in HP. Scale bar indicates 20 μm.

**Fig 4:**
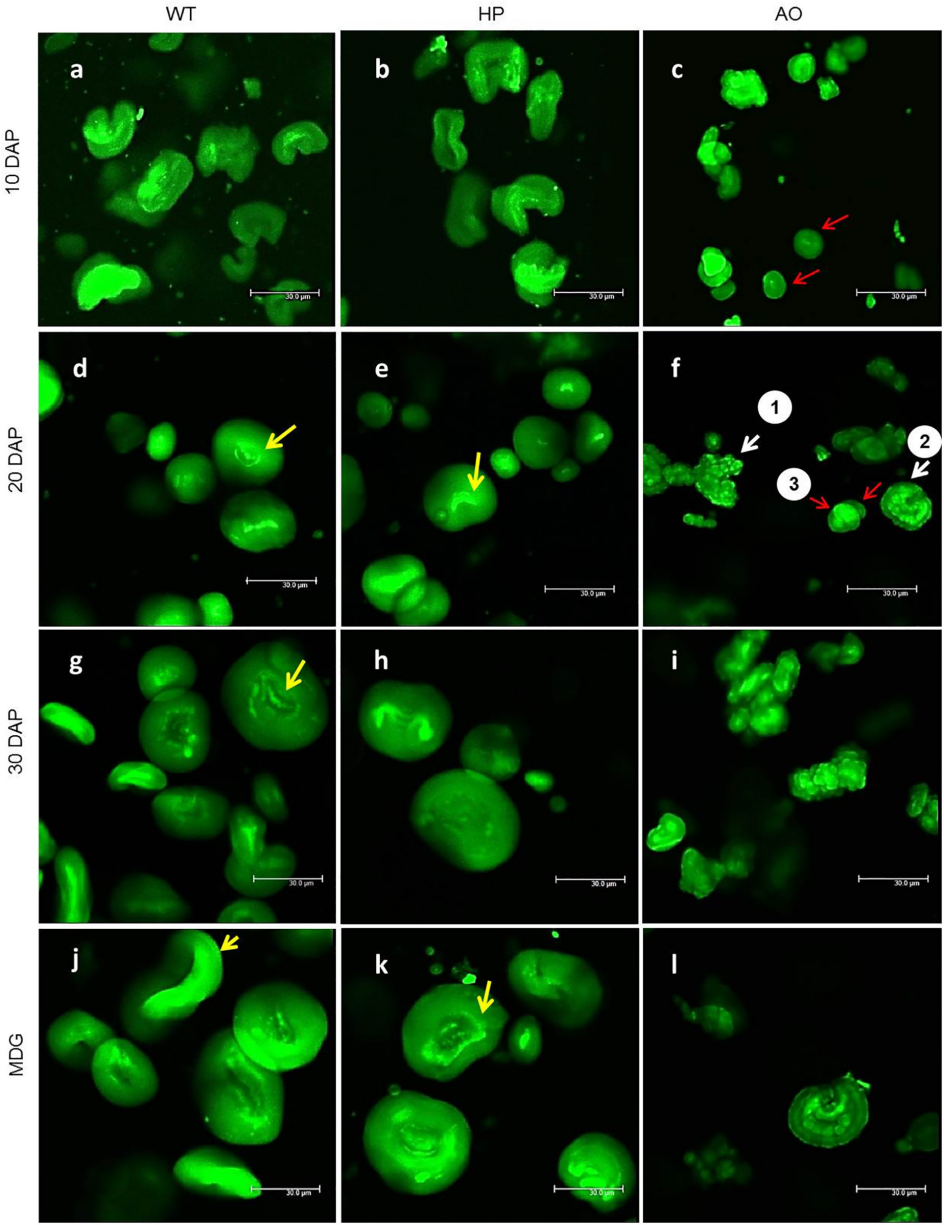
Confocal laser scanning micrographs showing inner structural details of purified starch granules stained with APTS. Images are from different developmental stages (10, 20, 30 DAP and MDG) at lower magnification of WT (a,d,g,j), HP (b,e,h,k) and AO (c, f, i, l). Arrows (yellow) show hila (d,e,g) and uni-concave A-type granules (j,k), normal granules in AO indicated by red arrows (c,f) and typical granules in AO (f) indicated by 1 (highly distorted and multi-lobed), 2 (uni-concave discoid with multi-lobed protrusions) and 3 (normal). Scale bar indicates 30 μm.

The starch granule micro- and nano-topography as studied by non-coated Scanning Electron Microscopy (SEM) revealed features typical for the granule types synthesised by the three barley lines. Our previous studies [21,33] used coated SEM to reveal the general features of the HP and AO starch granules at mature stage. We now investigate developmental dynamics in starch granule biogenesis in these genotypes using non-coated SEM significantly enhancing credibility and resolution of native structures of the specimens. Very minor differences in morphology or topology in the initial stages of development were found (Fig 3a, 3e and 3i) and the granules were round/flattened with a smooth surface. At later stages HP showed tendency to a more uneven surface and granule shape with few pores at the surface in some granules as indicated (Fig 3g and 3h) while AO showed highly distorted granules (Fig 3k and 3l). The general shape variation within the granule populations was also evident as visualised by CLSM (Fig 4).

#### Internal starch granule structures

To obtain more comprehensive inner structural details of the starch granules and relate it to the topographical features, we investigated the purified starch granules at different developmental stages by Confocal Laser Scanning Microscopy (CLSM). 8-amino-1,3,6-pyrenetrisulfonic acid (APTS) specifically reacts with the reducing ends of amylose and amylopectin. Each starch molecular entity, being amylose, amylopectin or any intermediate material, contains only one reducing end and as an effect, APTS intensely stains amylose due to the 100–1000 smaller molecular size of amylose as compared to amylopectin. At lower magnification, WT and HP granules showed bright central hila, which seems to be organized as semi-circle (not as a prominent strong central spot but organized as elongated and semi-circular regions). WT and HP also had both large and small granules (observed from 20 DAP, as were also observed by SEM) (WT: Fig 4a, 4d, 4g and 4j and HP: Fig 4b, 4e, 4h and 4k, corresponding to bright-field image S4 Fig). The large granules showed a typical uni-concave discoid structure in both WT and HP. On the contrary, AO showed two irregular starch granule structures—distorted and multi-lobed granule structures and discoid structures with multiple protrusions (which showed similar uni-concave feature), and also a few spherical granule populations (not as large as the larger granules seen in WT and HP). The degree of irregularity in granule shape increased throughout the development of the AO (Fig 4c, 4f, 4i and 4l). At higher magnification, concentric growth rings with alternating bright and dark regions, bright central hila and bright protein filled channels were observed at 30 DAP and in MDG in WT (S5a and S5d Fig) and dark channels (voids) were observed in HP (S5b, S5e and S6 Figs) which could be the inner extensions of the surface pores observed by SEM, and also bright equatorial grooves (not shown here). Such surface pores and internal channels have been identified and studied elsewhere [37–39]. The population of discoid multinucleated structures observed in AO at lower magnification were identified as lobed protrusions on uni-concave discoid shaped granules (S5c and S5f Fig).

### Effects of SBE suppression and GWD over-expression on grain metabolism

In order to track down possible caryopsis developmental metabolic effects due to restructured starch granules in the HP and AO lines, we analysed the metabolite levels at all four developmental stages in WT, AO and HP by GC-MS based metabolite profiling [40]. We identified a total of 42 polar metabolites providing relatively good coverage of the metabolic pathways of starch synthesis and degradation, glycolysis, the TCA cycle, and amino acid metabolism. Additionally sugar phosphates were spectrophotometrically determined since they are closely related to starch metabolism. Changes in the metabolite profiles indicated indirect metabolic effects of SBE suppression and GWD over-expression.

#### Sugar, sugar alcohol and sugar phosphate levels

**Fig 5:**
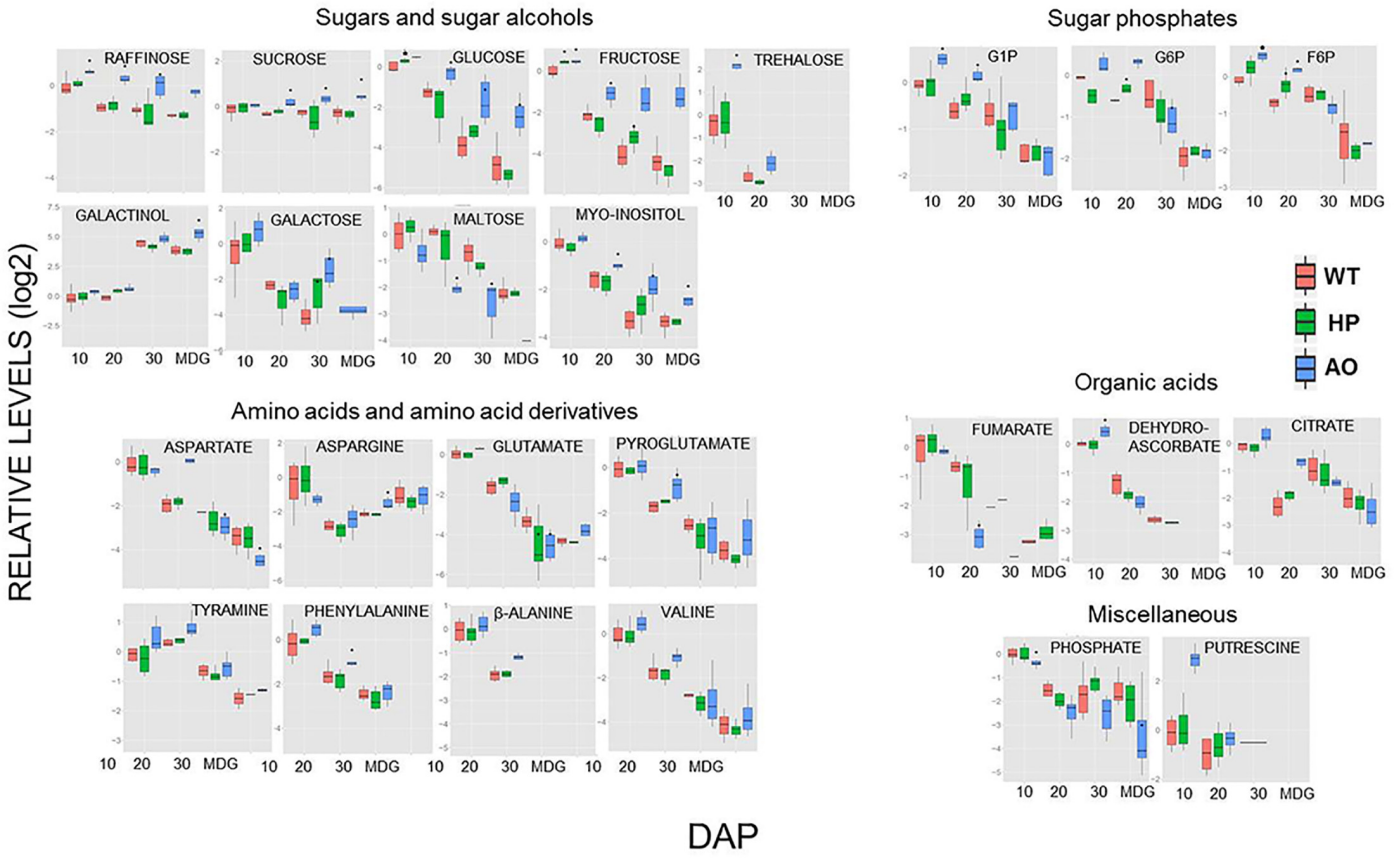
Relative levels of primary metabolites in the developing barley caryopsis of WT (red), HP (green) and AO (blue) at 10, 20, 30 DAP and MDG. The data is normalized to the WT 10 DAP sample and log2 transformed and presented as boxplots. The box and the horizontal line within the box represent the data at first and third quartile and median, respectively. The ends of vertical bars show maximum and minimum values. Student's *t*-test was performed to evaluate statistical difference between WT and transgenic plants for each time point by *t*-test. * indicates significant difference (P<0.05) to WT at each time point.

In all lines, sugar levels tended to decrease across development indicating their participation in starch synthesis (Fig 5, S7 Fig). Significant differences in sugars, sugar alcohols and sugar phosphates were observed in AO as compared to WT and HP. In general, the levels of sugars and sugar alcohols were higher in AO compared to WT. Higher levels of trehalose, raffinose, fructose, glucose, glucose-1-phosphate (G-1-P) and fructose-6-phosphate (F-6-P) were observed in AO while HP also showed higher fructose levels compared to WT at 10 DAP (Fig 5). At 20 DAP, higher levels of glucose, sucrose, raffinose, *myo*-inositol, G-1-P, glucose-6-phosphate (G-6-P) and F-6-P and lower levels of maltose were observed in AO while HP showed higher G-6-P and F-6-P levels than WT (Fig 5). At 30 DAP, AO showed higher levels of galactose, fructose, glucose, sucrose, raffinose, *myo*-inositol and lower levels of G-6-P and F-6-P while HP showed higher levels of galactose and fructose (Fig 5). At the mature stage, (MDG), higher levels of galactinol, raffinose, glucose and fructose were observed in AO compared to WT (Fig 5) demonstrating that sugar metabolites which is directly linked to starch biosynthesis are built up possibly due to partly blocked starch biosynthesis.

#### Levels of amino acids and their derivatives

The levels of amino acids were, in general, decreased over the development indicating their incorporation into storage protein (Fig 5, S7 Fig). No significant difference in the levels of amino acids were observed among the lines at 10 DAP other than a higher level of a diamine, putrescine in AO (Fig 5). At 20 DAP the levels of phenylalanine and aspartate were higher in AO compared to WT (Fig 5). At 30 DAP, AO showed lower levels of glutamate and aspartate, but higher levels of asparagine compared to WT (Fig 5). In MDG, AO showed lower levels of aspartate while HP showed no marked differences (Fig 5). Hence, again the AO showed more severe metabolic developmental changes than the HP.

#### Organic acid and phosphate levels

AT 10 DAP, AO showed higher level of dehydroascorbate and lower levels of the orthophosphate, while HP remained unaltered in the levels of organic acids at 10 DAP compared to WT (Fig 5). AO showed higher citrate and lower fumarate levels at 20 DAP (Fig 5). No marked differences in organic acids were observed in the lines in 30 DAP and at the mature stage (MDG) compared to WT.

### Principle Component Analysis (PCA)

**Fig 6:**
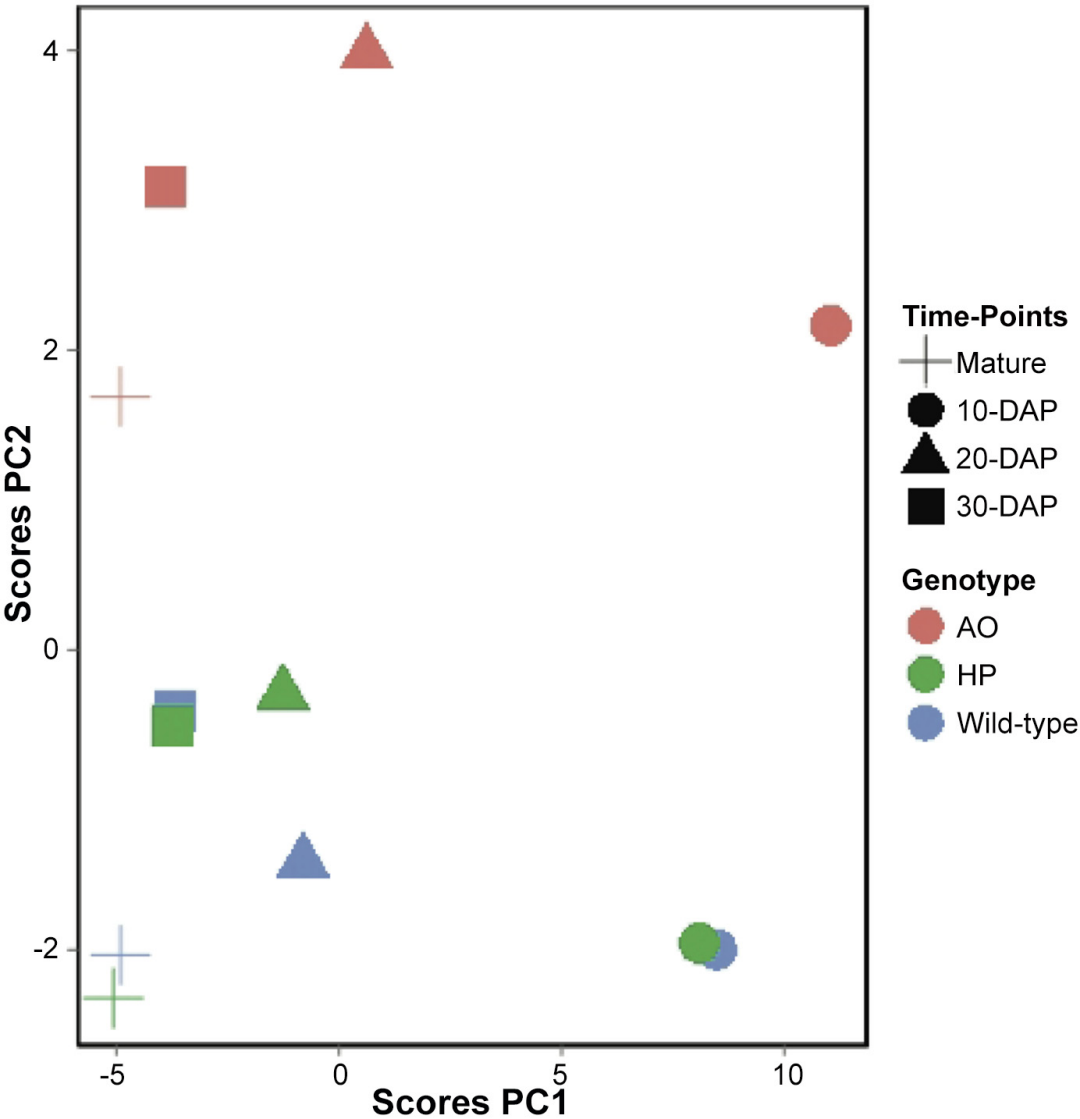
Principal component analysis of the metabolite profiles of grains during grain development. Each symbol represents the mean of all biological replicates (n = 6). The shape of symbols represents the time points. Cross, mature grain; circle, 10 days after pollination (DAP); triangle, 20 DAP; square, 30 DAP. The colours of the symbols are corresponding to the genotype. Red, AO line; green, HP line; blue, wild type.

In order to gain an overview of metabolite changes and grain composition, the main variation in the complete dataset was examined using PCA (Fig 6). At individual developmental stages, WT and HP were clustered together while AO is grouped separately (S8 Fig). At all developmental stages, the developmental stage differences fall on PC1 showing the highest variance (Fig 6), while the genotype differences lie along PC2 which showed WT and HP grouped together while AO is grouped separately away from both of these lines (Fig 6). The developmental stage differences were indicated mainly by the relocation of metabolites translated into storage reserve accumulation and showed greater variance than the genotype differences, which were contributed mostly by changes in sugar, sugar alcohols, polyamines and amino acids, and storage reserve accumulation.

## Discussion

### Re-direction of carbon partitioning in the grain

In previous studies we have described two *in planta* starch bioengineering techniques towards higher starch-bound phosphate content in the HP line [33] and an amylose-only starch type in the AO line [21]. Engineering metabolic pathways by targeting single enzymatic steps usually causes unintended pleiotropic effects that may often be deleterious. Monitoring such pleiotropic effects is therefore important for the evaluation of the individual *in planta* starch bioengineering approach. Our present study suggests that specific engineering of the starch biosynthesis, either directly or indirectly, affects carbon partitioning in the grain, depending on the targeted metabolic reaction. Generally we observed fewer pleiotropic effects in the HP line than in the AO line. The direct cause for the reduction of starch accumulation and increased sugar levels in the AO grains as compared to the WT and the HP grains is likely due to inefficient catalytic use of sugar precursors, because of limiting free non-reducing starch acceptor ends [41] in the mainly linear amylose as compared to the branched amylopectin. Similar effects have been observed in high-amylose systems e.g. in potato tubers [42]. An alternative explanation for the reduced starch biosynthesis rate could be that SBE, which is possibly involved in a multi-enzyme complex with accompanying starch synthesizing enzymes [12,43] is suggested to function to improve the efficiency of glucan polymer construction [11]. Absence of enzymes, such as SBE, in complexes can lead to a lack of coordination between these enzymes and thus lead to a reduced rate of biosynthesis. The low starch deposition rate can possibly itself explain some of the difference in phenotype observed in the AO line. In order to precisely assess the pure effects of starch structure on grain developmental metabolism isogenic lines with similar starch deposition rates must be compared. Such systems are however not trivial to obtain and other plant systems should be tested for that purpose.

Transient starch is synthesized in photosynthetic tissues during the day, and is degraded during night. Plants, which do not contain a functional GWD, such as the Arabidopsis *starch-excess 1* (*sex1)* mutant accumulate excess amounts of transient starch [44]. This suggests that the regulation of transient starch degradation is dependent on its phosphorylation by GWD. However, despite a diurnal variation in the gene expression of the GWD (SEX1) gene in Arabidopsis, the actual protein levels of GWD remain largely constant throughout the diurnal cycle [45]. The molecular structure of phospho-glucan suggests that phosphorylation by glucan kinases could be a steric prerequisite for stimulation of starch hydrolysis [8,35]. The role of starch phosphorylation in storage organs, such as tubers and seeds is less clear. Recent studies have shown that partial reduction of GWD expression in wheat grains does not change starch content [46]. In this study we over-expressed GWD (in the HP grains), and similarly did not observe any changes in starch content. The only minor topographical and morphological changes of the HP starch granules and the minor changes in sugar profiles compared to WT does not substantiate a major role of the elevated phosphorylation in barley grain starch biosynthesis.

The increased levels of BG and protein observed in the AO grains as compared to WT indicates a re-directed storage compound deposition from starch towards higher BG and protein biosynthesis as supported by the increase in specific amino acids in AO possibly related to the higher protein contents in the grain (Fig 2d). Similar effects have been reported in wheat (*Triticum aestivum*) [47–49] and pea seeds, (*Pisum sativum*), where deficiencies in seed starch biosynthesis resulted in excess accumulation of sugars, proteins and an altered water uptake [50–52]. Similarly, a shift from starch to BG accumulation has been observed in the low-starch *Lys5* barley mutant [53], which is deficient in the transport ADP—glucose transporter [54].

### Starch composition varied over grain development

The effects either of SBE silencing in the AO grains or the GWD over-expression in the HP grains seems to increase during grain development. In 10 DAP grains the amount of starch bound phosphate was around three times higher in HP, whereas it was around 10 times higher in MDG compared to the WT. Also, the presence of starch granule surface pores was specifically observed in HP only at the later in 30 DAP and MDG stages. This could be an effect of the hordein-D promoter, which is used in the overexpression of GWD in HP. This promoter activates storage protein accumulation, mostly at a later grain developmental stage than 10 DAP [33]. Similarly for the AO line, amylose content increased from below 80% of total starch at 10 DAP towards nearly 100% apparent amylose at grain maturity. The AO amylose was slightly branched, which is typical for normal amylose [36] and notably the intensity of the HPAEC-PAD chain detection between DP6 and DP60 was only 8.6% of that of the WT demonstrating the much reduced presence of short chains in the AO starch.

### Starch granules exhibited clear morphological differences over grain development

The starch granule topography of the HP starch granules in the maturation phase showed minute visual pores. The porous surface in the mature granules may be due to the hyper-phosphorylation causing enhanced hydration capacity and solubilization of starch amylopectin [55], which could stimulate attack by hydrolases present in the amyloplast during the maturation process, and in the endosperm, when cells undergo programmed cell death and disruption at the end of grain maturation [2].

Confocal images of starch granules in WT and HP at 10 DAP revealed contorted granules indicating lack of plasticity in the young granule structure [56], which eventually develop into mature discoid uni-concave granules. In AO, a few single spherical starch granules were observed in the initial developmental time points (10 and 20 DAP) but later (30 DAP and MDG) they disappeared. This may be a result of their fusions into the irregular compound mature granule, observed with higher frequency at these stages. Granule fusions have earlier been reported to occur between high-amylose starch granules, through double helical junctions among amylose surface chains of neighboring granules. Such fusions result in irregular compound granules that are enclosed by closely fitted sheath-like structure resembling pyrenoid starch sheaths [56].

### Effects on grain metabolism leading to osmotic and metabolic adjustments

Higher levels of sugar, sugar-phosphate and perturbation of amino acid metabolism were noted throughout development of AO grains. The lower starch accumulation in AO is associated with the accumulation of sugar-phosphates, and sugars like glucose, fructose and sucrose indicating the incapability to efficiently channelize these sugars into starch. Accumulation of raffinose and its precursor galactinol could be a feed forward effect of higher levels of G-6-P, G-1-P and F-6-P, which are precursors of their synthesis. Such, higher sugar and sugar alcohol levels in AO are likely to increase osmolarity and thereby water uptake and water content [57,58]. We have previously shown that AO grains have enlarged fluid-filled central cavities. This is a result of a deficient loading of sugars from the cavities into the endosperm cells, which causes excessive water to accumulate in the cavities [21]. Higher sucrose levels in AO can also cause re-partitioning of carbon to amino-acid biosynthesis via hormonal regulation and result in higher protein content. Hormonal regulation of C:N partitioning is observed during grain development in barley mutant deficient in cytosolic ADP-glucose pyrophosphorylase (AGPase). In this mutant sugars are over accumulated due to the inhibition of starch synthesis leading to the down regulation of amino acid synthesis, glycolysis and storage protein biosynthesis via the reduction of cytokinin levels [59]. Given the AO mutant also showed effects on starch and sugar accumulation, it is likely subjected to similar hormonal regulation. Interestingly protein biosynthesis is enhanced in AO mutant in contrast to the AGPase mutant. Since AGPase and SBE affects starch chain elongation and branching of amylose, respectively, differential regulations of protein synthesis indicate a possible role of polysaccharide composition or starch structure on the regulation of C:N partitioning.

It is also possible that the increase in sugars is a result of a stress response. Altered levels of citrate and fumarate indicate perturbation of respiratory metabolism in the early stage of grain development in AO. This likely leads to an accumulation of reactive oxygen species and results in the significant reduction of grain weight. Accumulation of compatible solutes including sucrose and raffinose under stress conditions has been reported in many studies [60] and considered as an adaptive response due to their roles in osmotic adjustments, antioxidative defense, protection of membrane and proteins [57,61–63]. The dehydroascorbate (DHA) is directly linked to the ascorbate level, which is regarded as a major biochemical indicator of oxidative stress given its role in scavenging reactive oxygen species (ROS). Accordingly, the higher levels of DHA found in AO at 10 DAP could indicate ROS accumulation as this compound accumulates under abiotic stress [64].

In conclusion, our data substantiates that minor modulation of the starch granule microstructure affecting its degree of phosphorylation (the HP line) has only minor effect on grain development. This is despite the suggested major metabolic importance of starch phosphorylation e.g. [46]. However directly modulating the microstructure and architecture of the starch granule (the AO line), results in a more fundamental metabolic re-direction and in the induction of stress-related metabolic pathways. Our study thus facilitates deeper understanding of starch structural effects on grain development and will as such permit further exploration of starch bioengineering strategies.

## Materials and Methods

### Plant growth and grain harvest

Barley line (*Hordeum vulgare* cv. Golden Promise, termed WT) and the two (T4) isogenic lines HP and AO, 25 plants / line, were cultivated in growth chambers under a light/dark regime of 16/8 h at 20/16°C and 70–75% of relative humidity (RH) at 200–250 μmol m^-2^ s^-1^ photon flux intensity at the plant level. The HP line is identical to the GWD-1 lines described in [33], and the AO line is identical to the SBE RNAi4.1 line described in [21]. The HP (GWD-1) line express the potato GWD1 driven by the endosperm specific hordein-D promoter and the AO line has simultaneous RNA-interference suppressed SBEI, SBEIIa and SBEIIb activities driven by the strong maize ubiquitin promoter. Determination of developmental stages for developing barley seeds were performed as described by [65]. The developing seeds were harvested from the middle region of the ear, starting from anthesis at 10, 20, 30 DAP until dry mature grain stage. Only six grains from each row were used for the present study. Since the metabolite levels in developing caryopsis are affected by the circadian rhythm, all the sampling was performed in the mid-light period to avoid any variations caused by diurnal adaptation of the caryopsis. Samples were immediately frozen in liquid nitrogen, freeze-dried and stored at -80°C until extraction.

### Grain growth parameters

The freeze-dried grain samples were homogenized in a tissue lyser (TissueLyser, Retsch, Qiagen) operating at 30 Hz for 15 s and passed through a 0.5 mm screen. The samples were stored at -80°C until analysis. Fresh weight, dry weight and water content were measured from a pool of 10 grains (harvested from the middle of the spike as was done for metabolite profiling) each with three biological replicates of WT, HP and AO. The fresh weight per grain was calculated and the grains were lyophilized for three days until they were completely dry and the weights measured again, which correspond to the dry weight and the loss of weight was calculated as the % of water content.

### Total carbon, total nitrogen and protein content

Total nitrogen and total carbon was determined on finely ground flour samples of five biological replicates each from WT, HP and AO, harvested at 10, 20, 30 DAP and MDG, using ANCA-SL/GSL elemental analyzer (SerCon, UK), employing the Dumas procedure for preparation and a stable isotope mass spectrometer. Nitrogen values were multiplied by a factor of 5.45 to give protein values.

### Total Starch content

Flour samples of the same five biological replicates used in the total carbon analysis, each from WT, HP and AO, harvested at 10, 20, 30 DAP and MDG were used for the analysis. Flour samples of 10 mg each were used and the analysis was performed as described previously [10].

### BG content

10 mg of flour samples of the same five biological replicates used in the total carbon analysis, each from WT, HP and AO harvested at 10, 20, 30 DAP and MDG were used for the analysis. BG content in the samples was determined exactly as described previously [10].

### Isolation of Starch

Isolation of starch was carried out from the flour samples of the same five biological replicates used in the total carbon analysis, each from WT, HP and AO, harvested at 10, 20, 30 DAP and MDG. Starch was extracted and purified as described previously [10].

### Starch granule size distribution

Starch granule size distribution was measured by Microtrac S3500 Particle Size Analyzer. The purified starch samples from the MDG of WT, HP and AO were dispersed in water and were subjected to mild ultra-sonication to aid proper dispersion of the starch granules and avoid agglomerates (especially in AO). The analysis was performed as per the instructions of the instrument.

### Amylose content by Iodine complexation

The amylose content was determined on the starch purified from five biological replicates each of WT, HP and AO at 10, 20, 30 DAP and MDG by iodine colorimetry as described previously [66].

### Starch bound C6 phosphate content

Starch bound C6 phosphate content was analyzed on the starch purified from the same five biological replicates, each of WT, HP and AO at 10 DAP and MDG. The degree of starch C6 phosphorylation was determined as the content of Glucose 6-P (G6P) after starch hydrolysis, using high- performance anion-exchange chromatography (Dionex, DX 500 system equipped with an S-3500 auto sampler, GP40 pump, ED40 PAD system fitted with a CarboPac PA-1 column) as previously described [67]. Standard curves for phosphate content analysis were based on characterized potato starches (potato, cv Dianella, [68].

### Determination of Average DP of debranched starch by HPAEC-PAD

Starch samples (5 mg/ml) from the same five biological replicates, each of WT, HP and AO at 10 DAP and MDG were gelatinized in water and enzymatically debranched at 40°C using 0.3 unit isoamylase (Megazyme, Sydney, Australia) per 1 mg of sample. The obtained linear glucan fragments were then analyzed using high pressure anion exchange chromatography with pulsed amperometric detection (HPAEC-PAD) by injecting 20 μl of sample on a CarboPac PA-200 column. The peaks corresponding to degree of polymerization (DP) 6 to 75 were integrated, corrected for detector response and the average DP was calculated as described in [69].

### Extraction, derivatization, and analysis of barley grain metabolites

Polar metabolites were extracted from 10 mg of lyophilized grain materials. Grains from six individual WT, HP and AO plants were harvested at 10, 20, 30 DAP and MDG. Metabolite extraction, derivatization, and gas chromatography time-of-flight mass spectrometry (GC-TOF-MS) analysis were performed [40]. Metabolites were manually identified using the reference library mass spectra and retention indices from the Golm Metabolome Database (GMD, http://gmd.mpimp-golm.mpg.de; [70]. Both chromatograms and mass spectra were evaluated using TAGFINDER [71]. The parameters used for the peak annotation are listed in S1 Tables according to [72]. A part of metabolite extract was used for the determination of Glucose-6-P (G-6-P), glucose-1-P (G-1-P) and fructose-6-P (F-6-P) using the method described by [73].

### APTS staining

Starch granules were stained with 8-amino-1,3,6-pyrenetrisulfonic acid (APTS) as described previously in [74]. Starch granules (~2 mg) were incubated in 3 μL APTS solution (20 mM, dissolved in 15% acetic acid) and an equal volume of 1 M sodium cyanoborohydride. The mixture was incubated at 30°C for 15–18 h. The granules were washed in distilled water and suspended in 20 μL of 50% glycerol. For microscopy, 1 μL of the granule preparation was fixed in a mixture of 2% agar and 80% glycerol in water and mounted onto a glass slide for microscopy.

### Confocal Laser Scanning Microscopy (CLSM)

Images of APTS stained starch granule were recorded on a confocal laser scanning microscope (TCS SP2, Leica Microsystems, Germany) as described in [74]. The confocal laser scanning microscope used was equipped with an argon laser. A laser power of 25% was maintained during acquisition of all APTS images, and the gain was varied to prevent saturation of the detector and to ensure comparable fluorescence intensities in all images. Due the high amylose content, the AO granules had intense fluorescence and as a result, detection gain was reduced to avoid saturation in AO micrographs. The following objectives and filter settings were used: 10 X dry objective and 40X plan apo/ 1.25–0.75 oil CS, excitation wavelength: 488 nm, beam splitter: TD 488/543/ 633, light was detected at the interval from 500 to 535 nm.

### Scanning Electron Microscopy

Non-coated images of purified starch granules were taken in an FEI Helios Nanolab600 dual beam, electron-ion microscope. The secondary electrons were collected with the Thru-the-Lens detector for a 43 pA incident electron beam accelerated with 1 Kev. The working distance was varied from 1.9 to 2.2 mm as needed for each individual sample. All samples were prepared by sonicating a minute amount of starch into 0.5 ml milliQ water for 5 min. Next a droplet of solution was placed onto a 1x1 cm^2^ piece of a silicon wafer, which was attached onto an aluminum stub with a double sided carbon tape. The excess of solution was dried out with a piece of filter paper, barely touching the side of the Silicon piece. The samples were further left for drying at room temperature in a plastic box until imaged.

### Statistical Analysis

The significance tests were performed using the one-way ANalysis Of VAriance (ANOVA) statistical tool embedded into SigmaPlot 12.0. The term significant is used in the text only when the change has been confirmed to be significant (P < 0.05/0.01/0.001) with the one-way ANOVA. For metabolite data, students t-test function in R was used to show the significant differences (P<0.05).

### Principal component analysis (PCA)

PCA was performed on data sets obtained from metabolite profiling and grain compositional analysis (starch, amylose, BG, protein) by the prcomb function in the R-software environment (http://cran.r-project.org/). The data was centered and auto-scaled before the analysis.

## Supporting Information

S1 Fig Comparison of morphological changes occurring in the developing barley caryopsis of WT, HP and AO. The developmental stages used for the study are 10, 20 and 30 Days After Pollination (DAP) and divided into two phases: storage phase and desiccation phase based on storage reserve accumulation and water loss. Single representative grains are shown for each genotype at each phase.
(DOCX)

S2 Fig Difference amylopectin chain length distributions plot of starch from 10 DAP (top panel) and mature starch samples (bottom panel). WT chain length distribution (zero value line) as compared to HP and AO. Distributions were standardised to 100% for all three samples and the amount of liberated chains from the AO starch was only 8% of the WT and HP, respectively.
(DOCX)

S3 Fig Starch granule size distribution of WT, HP and AO purified starches from MDG stage. The purified starch samples from the MDG of WT, HP and AO are dispersed in water and are subjected to ultrasonication to aid proper dispersion of the starch granules and avoid agglomerates. No obvious difference was found between HP and WT but AO showed reduction in the first peak appearing before 10 μm indicating lesser small granules (<10 μm) and is slightly shifted to the farther side of the peak indicating larger granule size which can be due to the typical structures observed in AO.
(DOCX)

S4 Fig Bright—field images of the starch granules shown in confocal laser scanning micrographs (Fig 4). Scale bar indicates 30 μm.
(DOCX)

S5 Fig Confocal laser scanning micrographs showing inner structural details of purified starch granules stained with APTS. Images are from different developmental stages (10, 20, 30 DAP and MDG) at higher magnification of WT (a,d), HP (b,e) and AO (c,f). Arrows indicate locations of growth rings (yellow), bright and dark channels (red), and typical protrusions on AO (white) of starch granules. Scale bar indicates 20 μm.
(DOCX)

S6 Fig Confocal laser scanning micrograph of HP starch granules indicating protein filled bright channels in purified starch granules stained with APTS. Scale bar indicates 20 μm.
(DOCX)

S7 Fig Primary metabolite levels of all analysed metabolites in the developing barley caryopsis of WT, HP and AO at 10, 20, 30 DAP and MDG. Data are normalized to the internal standard (ribitol). Values are means of ± SE of six biological replicates. VANTED (Björn et al 2006) is used for visualization. Asterisks indicate significant differences (* denotes P<0.05) performed by t-test embedded in VANTED.
(DOCX)

S8 Fig Principal component analysis of the metabolite profiles of grains at each time point of grain development. Each symbol represents the value of each biological replicate (n = 6). The colours of the symbols are corresponding to the genotype. Red, AO line; green, HP line; blue, wild type.
(DOCX)

S1 Tables Metabolite Reporting Guidelines (Checklist Table) and Overview Table of the metabolite reporting list. (XLSX)
