# Supplementary material for: Starch Granule Re-Structuring by Starch Branching Enzyme and Glucan Water Dikinase Modulation Affects Caryopsis Physiology and Metabolism
Source: PLoS One. 2016 Feb 18;11(2):e0149613. doi: 10.1371/journal.pone.0149613 (PMC4758647; doi:10.1371/journal.pone.0149613)
Supplement: S1 Fig — The developmental stages used for the study are 10, 20 and 30 Days After Pollination (DAP) and divided into two phases: storage phase and desiccation phase based on storage reserve accumulation and water loss. Single representative grains are shown for each genotype at each phase. (DOCX) [file pone.0149613.s001.docx]

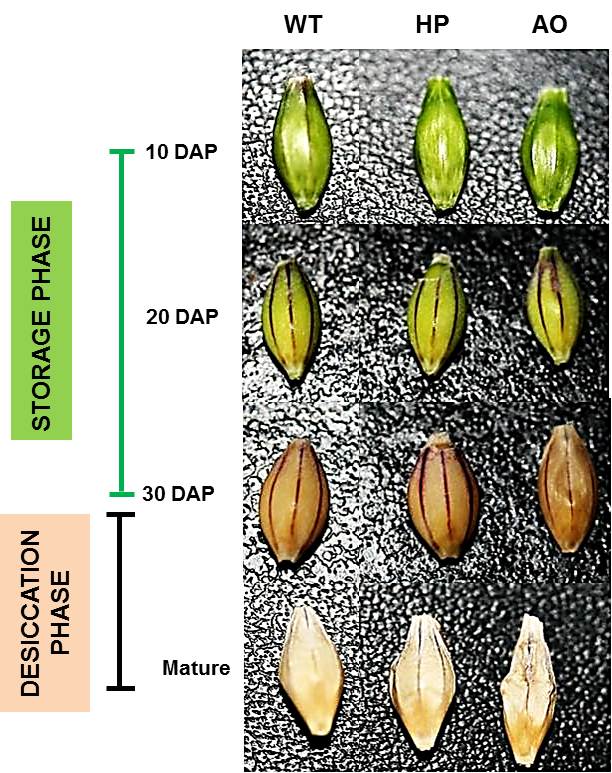


**S1 Fig.** Comparison of morphological changes occurring in the developing barley caryopsis of WT, HP and AO. The developmental stages used for the study are 10, 20 and 30 Days After Pollination (DAP) and divided into two phases: storage phase and desiccation phase based on storage reserve accumulation and water loss.
