# Supplementary material for: Starch Granule Re-Structuring by Starch Branching Enzyme and Glucan Water Dikinase Modulation Affects Caryopsis Physiology and Metabolism
Source: PLoS One. 2016 Feb 18;11(2):e0149613. doi: 10.1371/journal.pone.0149613 (PMC4758647; doi:10.1371/journal.pone.0149613)
Supplement: S2 Fig — WT chain length distribution (zero value line) as compared to HP and AO. Distributions were standardised to 100% for all three samples and the amount of liberated chains from the AO starch was only 8% of the WT and HP, respectively. (DOCX) [file pone.0149613.s002.docx]

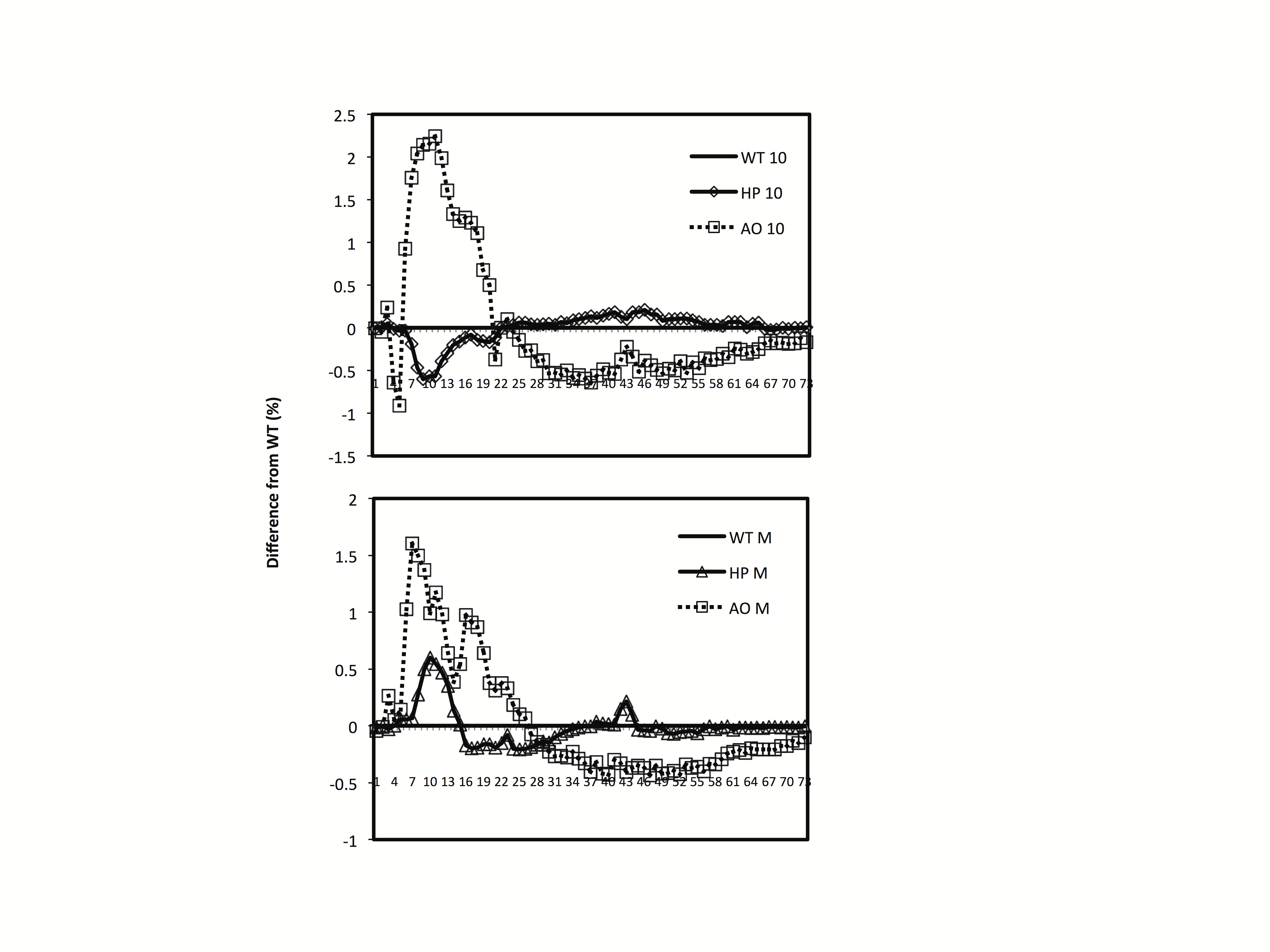


**S2 Fig.** Difference amylopectin chain length distributions plot of starch from 10 DAP (top panel) and mature starch samples (bottom panel). WT chain length distribution (zero value line) as compared to HP and AO. Distributions were standardised to 100% for all three samples and the amount of liberated chains from the AO starch was only 8% of the WT and HP, respectively.
