# Supplementary material for: Starch Granule Re-Structuring by Starch Branching Enzyme and Glucan Water Dikinase Modulation Affects Caryopsis Physiology and Metabolism
Source: PLoS One. 2016 Feb 18;11(2):e0149613. doi: 10.1371/journal.pone.0149613 (PMC4758647; doi:10.1371/journal.pone.0149613)
Supplement: S3 Fig — The purified starch samples from the MDG of WT, HP and AO are dispersed in water and are subjected to ultrasonication to aid proper dispersion of the starch granules and avoid agglomerates. No obvious difference was found between HP and WT but AO showed reduction in the first peak appearing before 10 μm indicating lesser small granules (<10 μm) and is slightly shifted to the farther side of the peak indicating larger granule size which can be due to the typical structures observed in AO. (DOCX) [file pone.0149613.s003.docx]

**S3 Fig.** Starch granule size distribution of WT, HP and AO purified starches from MDG stage. The purified starch samples from the MDG of WT, HP and AO are dispersed in water and are subjected to ultrasonication to aid proper dispersion of the starch granules and avoid agglomerates. No obvious difference was found between HP and WT but AO showed reduction in the first peak appearing before 10 µm indicating lesser small granules (<10 µm) and is slightly shifted to the farther side of the peak indicating larger granule size which can be due to the typical structures observed in AO.
