# Supplementary material for: Starch Granule Re-Structuring by Starch Branching Enzyme and Glucan Water Dikinase Modulation Affects Caryopsis Physiology and Metabolism
Source: PLoS One. 2016 Feb 18;11(2):e0149613. doi: 10.1371/journal.pone.0149613 (PMC4758647; doi:10.1371/journal.pone.0149613)
Supplement: S4 Fig — Scale bar indicates 30 μm. (DOCX) [file pone.0149613.s004.docx]

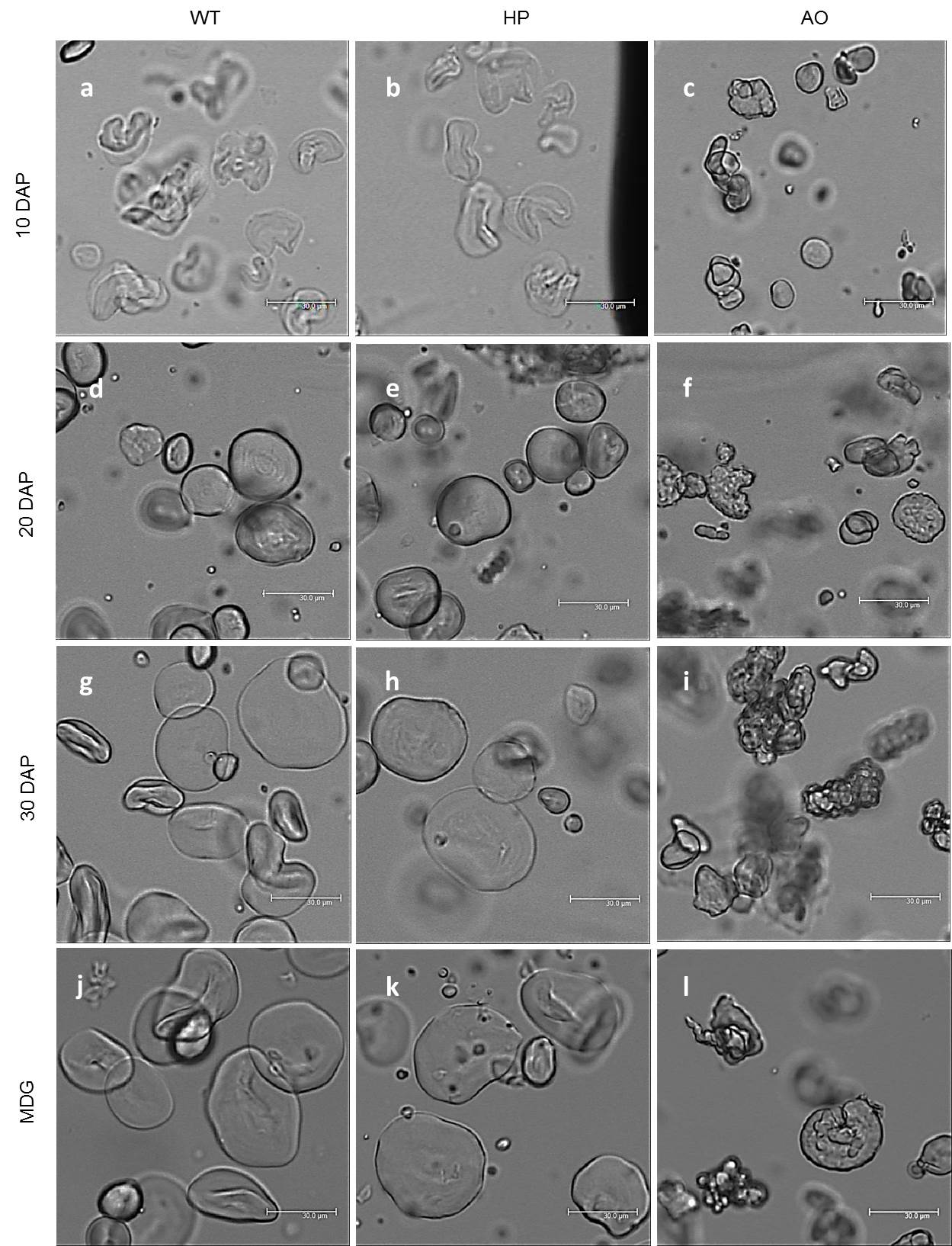


**S4 Fig.** Bright –field images of the starch granules shown in confocal laser scanning micrographs (Fig. 4). Scale bar indicates 30 µm.
