# Supplementary material for: Starch Granule Re-Structuring by Starch Branching Enzyme and Glucan Water Dikinase Modulation Affects Caryopsis Physiology and Metabolism
Source: PLoS One. 2016 Feb 18;11(2):e0149613. doi: 10.1371/journal.pone.0149613 (PMC4758647; doi:10.1371/journal.pone.0149613)
Supplement: S5 Fig — Images are from different developmental stages (10, 20, 30 DAP and MDG) at higher magnification of WT (a,d), HP (b,e) and AO (c,f). Arrows indicate locations of growth rings (yellow), bright and dark channels (red), and typical protrusions on AO (white) of starch granules. Scale bar indicates 20 μm. (DOCX) [file pone.0149613.s005.docx]

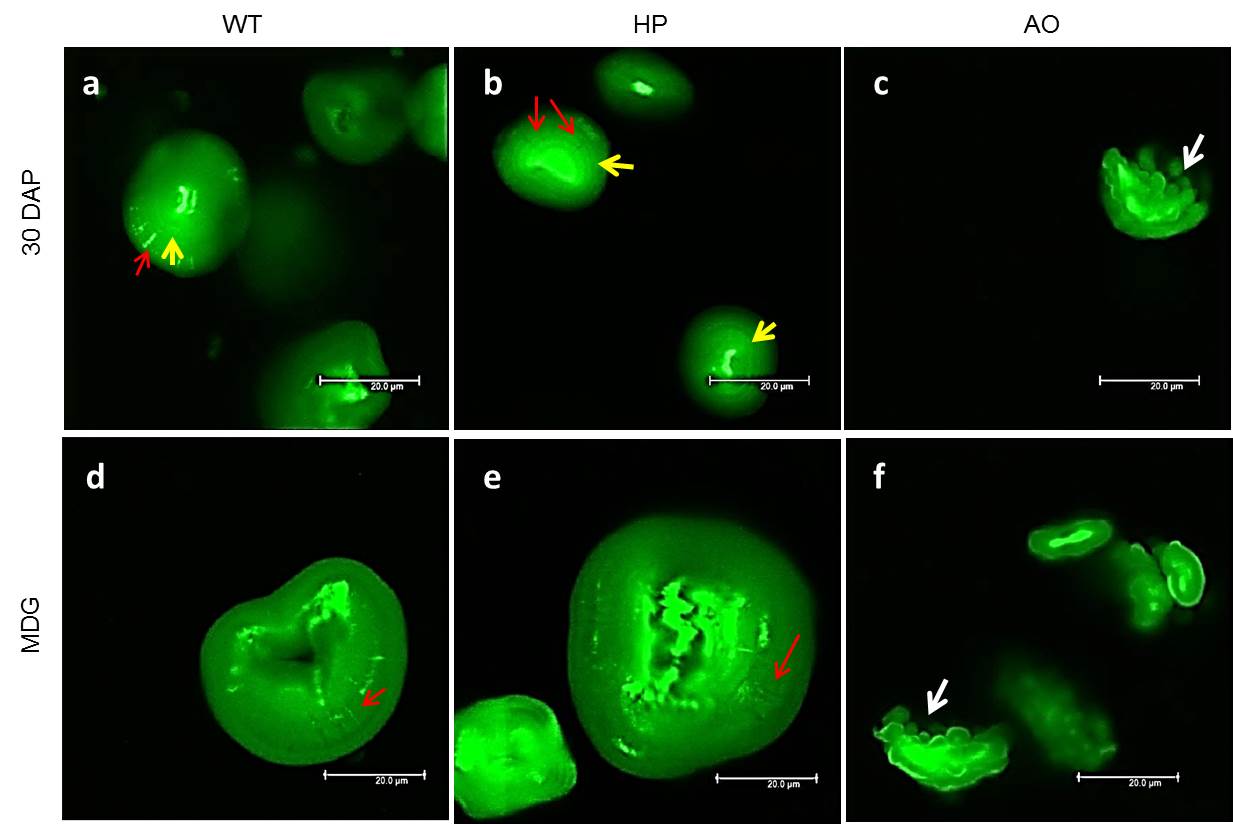


**S5 Fig.** Confocal laser scanning micrographs showing inner structural details of purified starch granules stained with APTS. Images are from different developmental stages (10, 20, 30 DAP and MDG) at higher magnification of WT (a,d), HP (b,e) and AO (c,f). Arrows indicate locations of growth rings (yellow), bright and dark channels (red), and typical protrusions on AO (white) of starch granules. Scale bar indicates 20 µm.
