# Supplementary material for: Starch Granule Re-Structuring by Starch Branching Enzyme and Glucan Water Dikinase Modulation Affects Caryopsis Physiology and Metabolism
Source: PLoS One. 2016 Feb 18;11(2):e0149613. doi: 10.1371/journal.pone.0149613 (PMC4758647; doi:10.1371/journal.pone.0149613)
Supplement: S6 Fig — Scale bar indicates 20 μm. (DOCX) [file pone.0149613.s006.docx]

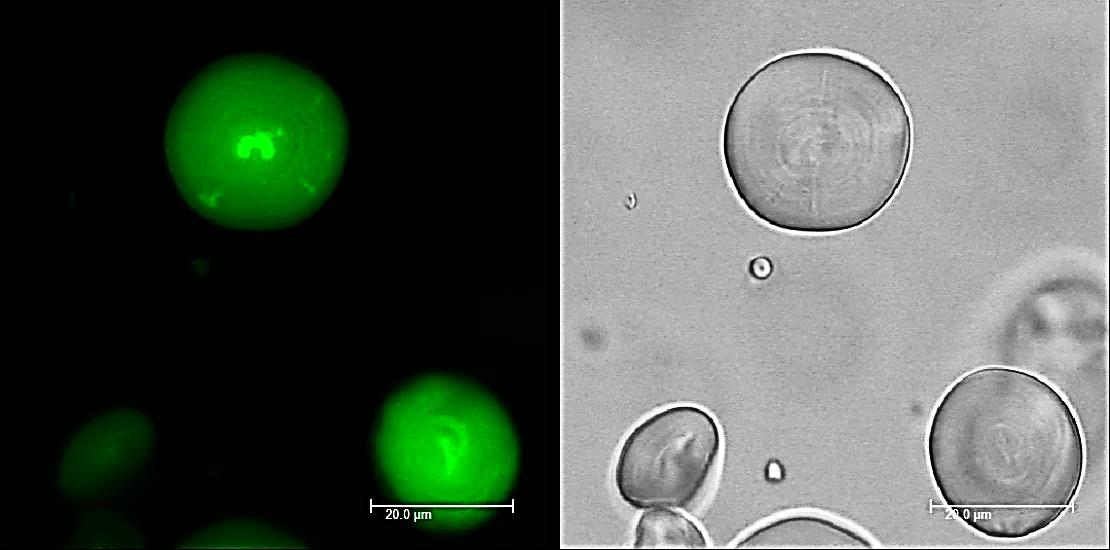


**S6 Fig.** Confocal laser scanning micrograph of HP starch granules indicating protein filled bright channels in purified starch granules stained with APTS. Scale bar indicates 20 µm.
