# Supplementary material for: Starch Granule Re-Structuring by Starch Branching Enzyme and Glucan Water Dikinase Modulation Affects Caryopsis Physiology and Metabolism
Source: PLoS One. 2016 Feb 18;11(2):e0149613. doi: 10.1371/journal.pone.0149613 (PMC4758647; doi:10.1371/journal.pone.0149613)
Supplement: S7 Fig — Data are normalized to the internal standard (ribitol). Values are means of ± SE of six biological replicates. VANTED (Björn et al 2006) is used for visualization. Asterisks indicate significant differences (* denotes P<0.05) performed by t-test embedded in VANTED. (DOCX) [file pone.0149613.s007.docx]

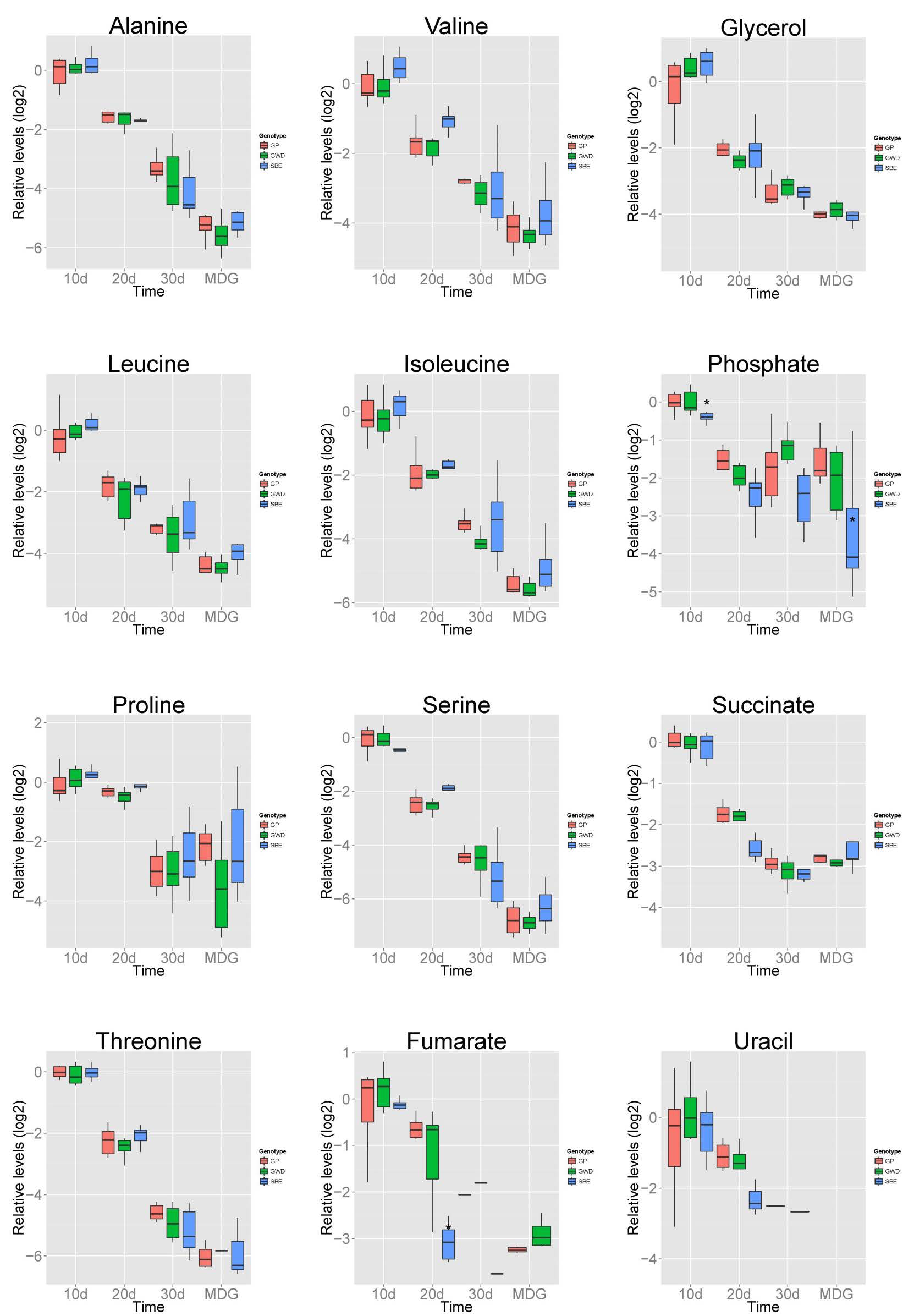


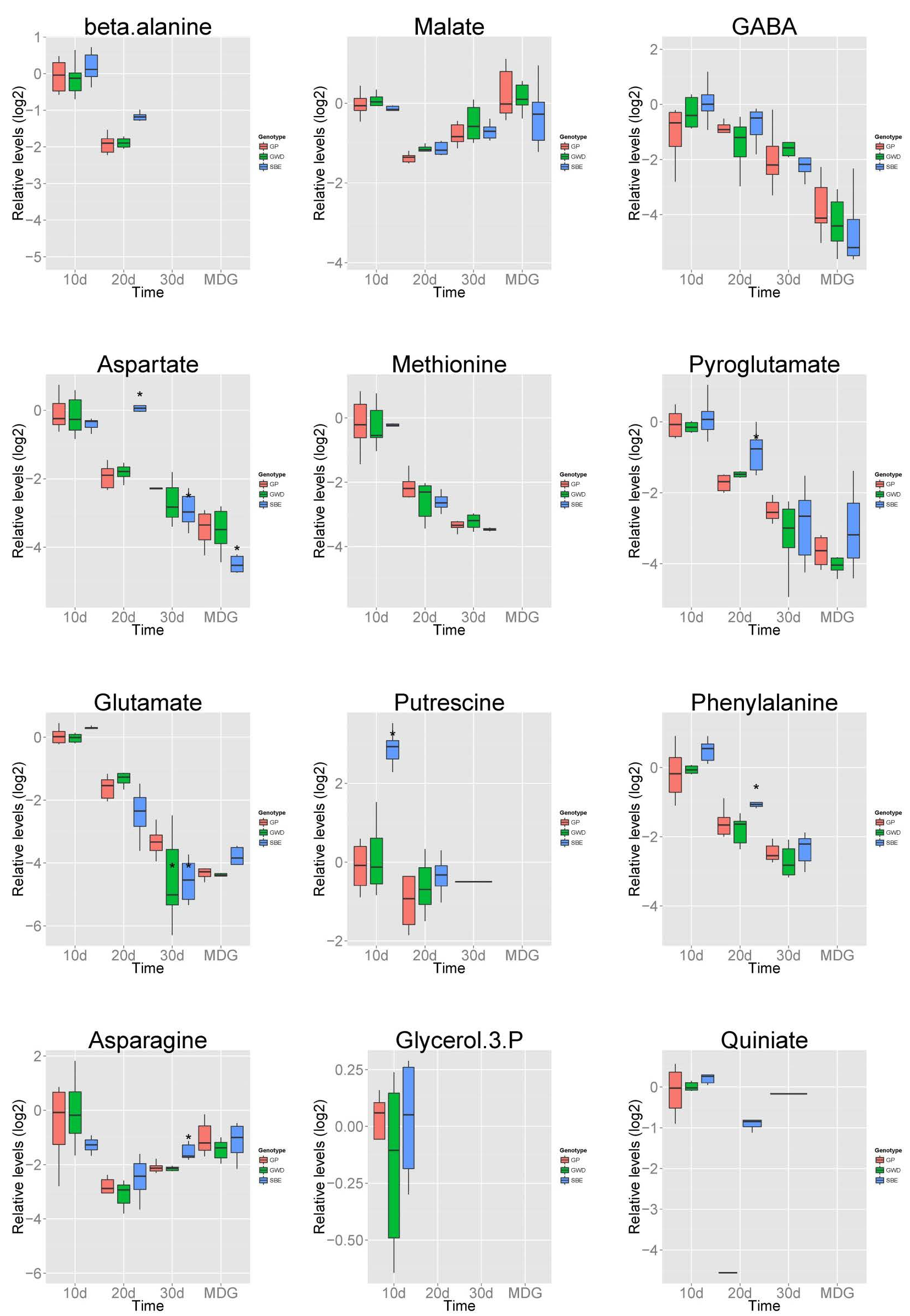


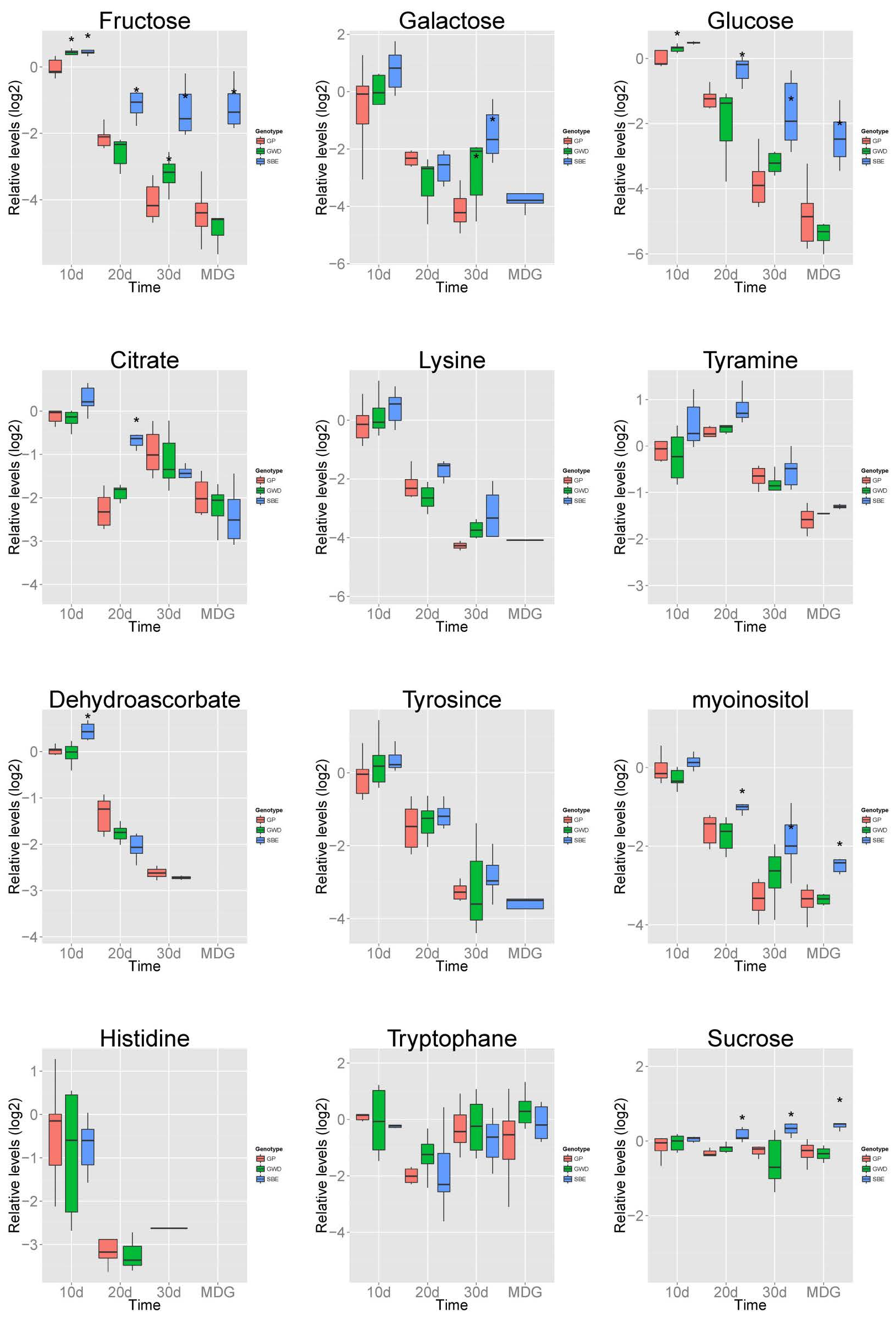


**
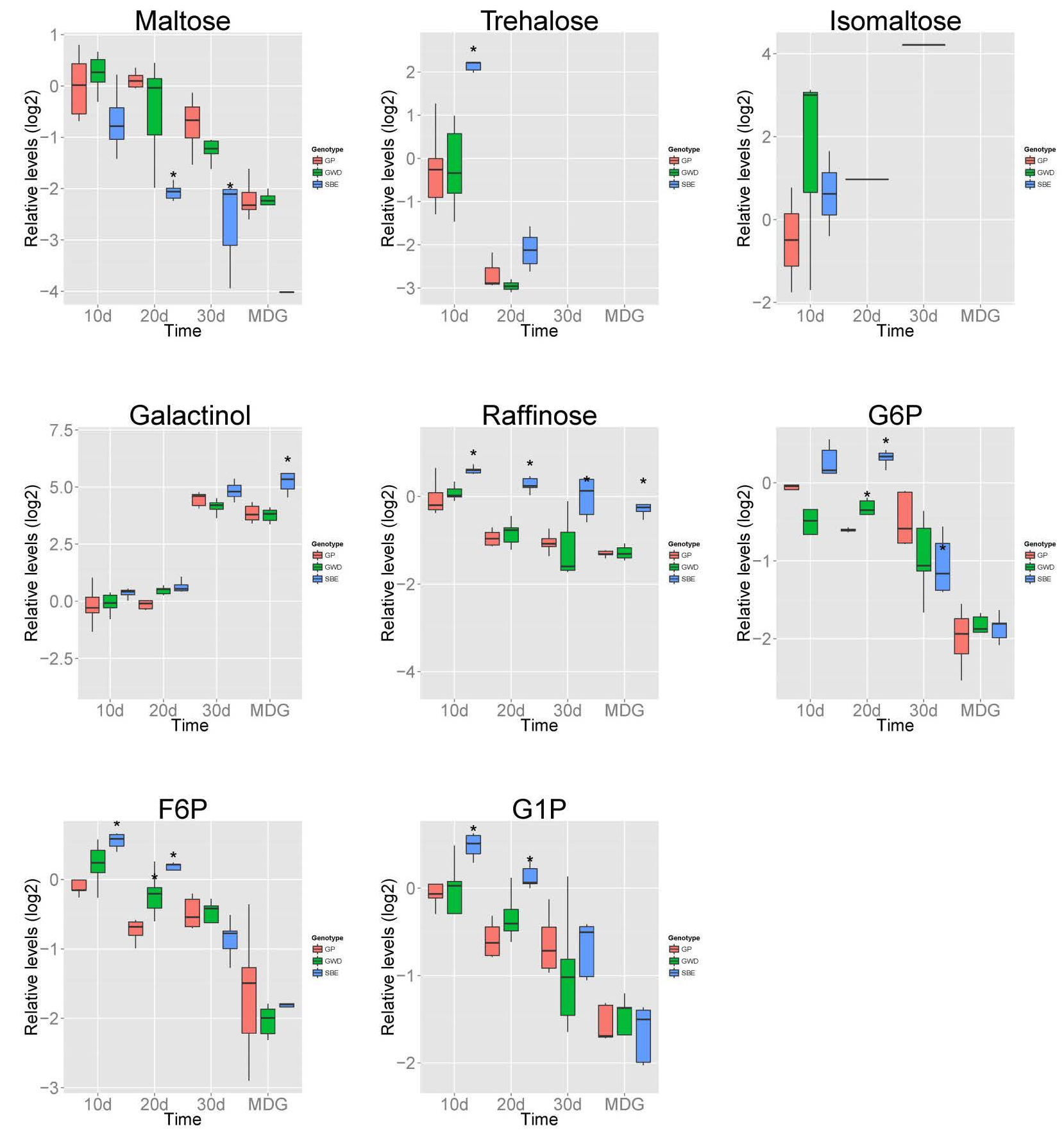
**

**S7 Fig.** Primary metabolite levels of all analysed metabolites in the developing barley caryopsis of WT, HP and AO at 10, 20, 30 DAP and MDG. Data are normalized to the internal standard (ribitol). Values are means of ± SE of six biological replicates. VANTED (Björn et al 2006) is used for visualization. Asterisks indicate significant differences (* denotes P<0.05) performed by t-test embedded in VANTED.
