# Supplementary material for: Starch Granule Re-Structuring by Starch Branching Enzyme and Glucan Water Dikinase Modulation Affects Caryopsis Physiology and Metabolism
Source: PLoS One. 2016 Feb 18;11(2):e0149613. doi: 10.1371/journal.pone.0149613 (PMC4758647; doi:10.1371/journal.pone.0149613)
Supplement: S8 Fig — Each symbol represents the value of each biological replicate (n = 6). The colours of the symbols are corresponding to the genotype. Red, AO line; green, HP line; blue, wild type. (DOCX) [file pone.0149613.s008.docx]

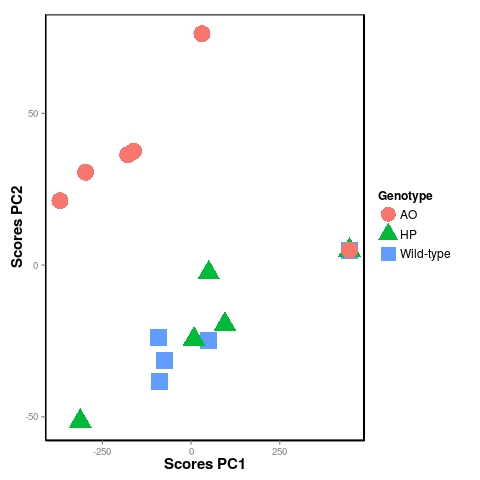

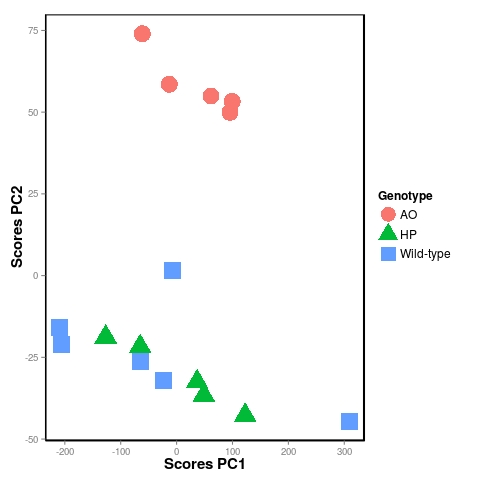

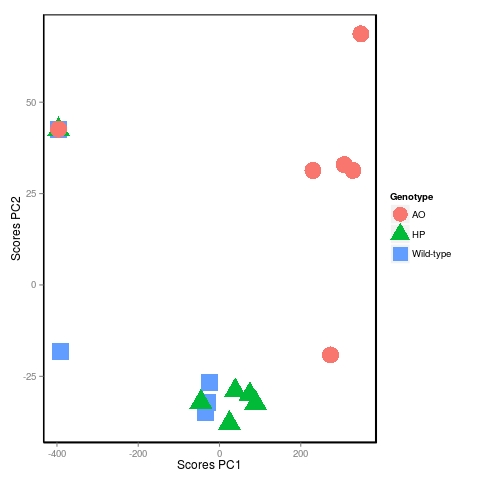

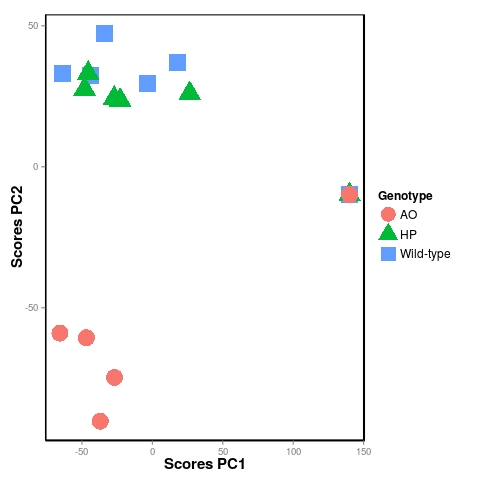


(d) Mature

(a) 10 DAP

(b) 20 DAP

(c) 30DAP

**S8 Fig.** Principal component analysis of the metabolite profiles of grains at each time point of grain development. Each symbol represents the value of each biological replicate (n=6). The colours of the symbols are corresponding to the genotype. Red, AO line; green, HP line; blue, wild type.
